# Innovative approaches for cancer treatment: current perspectives and new challenges

**DOI:** 10.3332/ecancer.2019.961

**Published:** 2019-09-10

**Authors:** Carlotta Pucci, Chiara Martinelli, Gianni Ciofani

**Affiliations:** 1 Smart Bio-Interfaces, Istituto Italiano di Tecnologia, 56025 Pisa, Italy; 2 Department of Mechanical and Aerospace Engineering, Politecnico di Torino, 10129 Torino, Italy; a https://orcid.org/0000-0002-8976-3711; b https://orcid.org/0000-0001-9360-1689; c https://orcid.org/0000-0003-1192-3647

**Keywords:** cancer, nanomedicine, extracellular vesicles, targeted therapy, immunotherapy, gene therapy, thermal ablation, radiomics, pathomics

## Abstract

Every year, cancer is responsible for millions of deaths worldwide and, even though much progress has been achieved in medicine, there are still many issues that must be addressed in order to improve cancer therapy. For this reason, oncological research is putting a lot of effort towards finding new and efficient therapies which can alleviate critical side effects caused by conventional treatments. Different technologies are currently under evaluation in clinical trials or have been already introduced into clinical practice. While nanomedicine is contributing to the development of biocompatible materials both for diagnostic and therapeutic purposes, bioengineering of extracellular vesicles and cells derived from patients has allowed designing *ad hoc* systems and univocal targeting strategies. In this review, we will provide an in-depth analysis of the most innovative advances in basic and applied cancer research.

## Introduction

Cancer is one of the main causes of death worldwide, and in the past decade, many research studies have focused on finding new therapies to reduce the side effects caused by conventional therapies.

During cancer progression, tumours become highly heterogeneous, creating a mixed population of cells characterised by different molecular features and diverse responsivity to therapies. This heterogeneity can be appreciated both at spatial and temporal levels and is the key factor responsible for the development of resistant phenotypes promoted by a selective pressure upon treatment administration [1]. Usually, cancer is treated as a global and homogeneous disease and tumours are considered as a whole population of cells. Thus, a deep understanding of these complex phenomena is of fundamental importance in order to design precise and efficient therapies.

Nanomedicine offers a versatile platform of biocompatible and biodegradable systems that are able to deliver conventional chemotherapeutic drugs *in vivo*, increasing their bioavailability and concentration around tumour tissues, and improving their release profile [2]. Nanoparticles can be exploited for different applications, ranging from diagnosis to therapy [2].

Recently, extracellular vesicles (EVs), responsible for cancer development, microenvironment modification and required for metastatic progression, have been widely investigated as efficient drug delivery vehicles [3].

Natural antioxidants and many phytochemicals have been recently introduced as anti-cancer adjuvant therapies due to their anti-proliferative and pro-apoptotic properties [4, 5].

Targeted therapy is another branch of cancer therapy aiming at targeting a specific site, such as tumour vasculature or intracellular organelles, leaving the surroundings unaffected. This enormously increases the specificity of the treatment, reducing its drawbacks [6].

Another promising opportunity relies on gene therapy and expression of genes triggering apoptosis [7] and wild type tumour suppressors [8], or the targeted silencing mediated by siRNAs, currently under evaluation in many clinical trials worldwide [9].

Thermal ablation of tumours and magnetic hyperthermia are opening new opportunities for precision medicine, making the treatment localised in very narrow and precise areas. These methods could be a potential substitute for more invasive practices, such as surgery [10, 11].

Furthermore, new fields such as radiomics and pathomics are contributing to the development of innovative approaches for collecting big amounts of data and elaborate new therapeutic strategies [12, 13] and predict accurate responses, clinical outcome and cancer recurrence [14–16].

Taken all together, these strategies will be able to provide the best personalised therapies for cancer patients, highlighting the importance of combining multiple disciplines to get the best outcome.

In this review, we will provide a general overview of the most advanced basic and applied cancer therapies, as well as newly proposed methods that are currently under investigation at the research stage that should overcome the limitation of conventional therapies; different approaches to cancer diagnosis and therapy and their current status in the clinical context will be discussed, underlining their impact as innovative anti-cancer strategies.

## Nanomedicine

Nanoparticles are small systems (1–1,000 nm in size) with peculiar physicochemical properties due to their size and high surface-to-volume ratio [17]. Biocompatible nanoparticles are used in cancer medicine to overcome some of the issues related to conventional therapies, such as the low specificity and bioavailability of drugs or contrast agents [2]. Therefore, encapsulation of the active agents in nanoparticles will increase their solubility/biocompatibility, their stability in bodily fluids and retention time in the tumour vasculature [18–20]. Furthermore, nanoparticles can be engineered to be extremely selective for a precise target [21, 22] (see the “Targeted therapy and immunotherapy” section) and to release the drug in a controlled way by responding to a specific stimulus [18, 23–25]. This is the case of ThermoDox, a liposomal formulation that can release doxorubicin as a response to an increment of temperature [26].

Inorganic nanoparticles are generally used as contrast agents for diagnosis purposes. Among them, quantum dots are small light-emitting semiconductor nanocrystals with peculiar electronic and optical properties, which make them highly fluorescent, resistant to photobleaching and sensitive for detection and imaging purposes [27]. Combined with active ingredients, they can be promising tools for theranostic applications [27]. In a recent study, quantum dots coated with poly(ethylene glycol) (PEG) were conjugated to anti-HER2 antibody and localised in specific tumour cells [28].

Superparamagnetic iron oxide nanoparticles (SPIONs) are usually exploited as contrast agents in magnetic resonance imaging (MRI) because they interact with magnetic fields [29, 30]. Five types of SPIONs have been tested for MRI: ferumoxides (Feridex in the US, Endorem in Europe), ferucarbotran (Resovist), ferucarbotran C (Supravist, SHU 555 C), ferumoxtran-10 (Combidex) and NC100150 (Clariscan). Ferucarbotran is currently available in few countries, while the others have been removed from the market [25]. SPIONs have also been studied for cancer treatment by magnetic hyperthermia (see the “Thermal ablation and magnetic hyperthermia” section), and a formulation of iron oxide coated with aminosilane called Nanotherm has been already approved for the treatment of glioblastoma [31].

Gold nanoparticles have raised interest because of their optical and electrical properties and low toxicity [32–34]. They are mainly used as contrast agents for X-ray imaging, computed tomography [25], photoacoustic imaging [35] and photodynamic therapy [36]. A nanoshell made of a silica core and a gold shell coated with PEG was approved by the Food and Drug Administration (FDA) in 2012 and commercialised as AuroShell (Nanospectra) for the treatment of breast cancer by photodynamic therapy [25].

Organic nanoparticles are mainly used as delivery systems for drugs. Liposomes and micelles are both made of phospholipids, but they differ in their morphology. Liposomes are spherical particles having at least one lipid bilayer, resembling the structure of cell membranes. They are mainly used to encapsulate hydrophilic drugs in their aqueous core, but hydrophobic drugs can also be accommodated in the bilayer or chemically attached to the particles [37]. Micelles, instead, own a hydrophobic core that can encapsulate hydrophobic drugs [38]. Doxil, doxorubicin-loaded PEGylated liposomes, were the first nanoparticles approved by the FDA in 1995 to treat AIDS-associated Kaposi’s sarcoma [39]. This formulation drastically reduces doxorubicin side effects. Since then, other liposomal formulations have been approved by the FDA for cancer therapy, such as Myocet and DaunoXome [40–42]. Polymeric nanoparticles are made of biocompatible or natural polymers, such as poly(lactide-co-glycolide), poly(ε-caprolactone), chitosan, alginate and albumin [43]. Some formulations have already been accepted by the FDA, such as Abraxane (albumin-paclitaxel particles for the treatment of metastatic breast cancer and pancreatic ductal adenocarcinoma) and Ontak (an engineered protein combining interleukin-2 and diphtheria toxins for the treatment of non-Hodgkin’s peripheral T-cell lymphomas).

As well as these systems, which have been either accepted or are under clinical investigation, it is worth mentioning some new nanoparticles currently undergoing testing at the research level, which should improve treatment performance. For example, solid lipid nanoparticles, made of lipids that are solid at body temperature [44], and fabricated to load hydrophobic drugs [45] have been demonstrated to give a higher drug stability and prolonged release compared to other systems; however, the encapsulation efficiency is often low because of their high crystallinity [46]. To overcome this issue, one or more lipids, liquid at room temperature (like oleic acid, for example), are included in the formulation [47]. Lipid nanoparticles are good candidates for brain tumour therapy as they are able to cross the blood–brain barrier (BBB) [48]. A recent work showed that lipid nanoparticles loaded with SPIONs and temozolomide are efficient to treat glioblastoma since they combine the effect of the conventional chemotherapy and hyperthermia [49, 50]. Dendrimers are another family of nanoparticles composed of polymers with a repetitive branched structure and characterised by a globular morphology [51, 52]. Their architecture can be easily controlled, making their structure extremely versatile for many applications. For example, some recent studies show that poly-L-lysine (PLL) dendrimers loaded with doxorubicin induce anti-angiogenic responses in *in vivo* tumour models [53]. Currently, there is only one clinical trial for a formulation named ImDendrim based on a dendrimer and on a rhenium complex coupled to an imidazolium ligand, for the treatment of inoperable liver cancers that do not respond to conventional therapies [54].

## Extracellular vesicles for cancer diagnosis and therapy

EVs are classified in two categories based on their biogenesis. Specifically, exosomes are small vesicles of around 30–150 nm originated from endosomes in physiological and pathological conditions and released by a fusion of multivesicular bodies (MVBs) to the cell membrane [55, 56], while shed microvesicles (sMVs), with a typical size of 50–1,300 nm, are present in almost any extracellular bodily fluid and are responsible for the exchange of molecular materials between cells [57, 58]. Exosomes are involved in cancer development and spreading [3, 59, 60], in the bidirectional communication between tumour cells and surrounding tissues, and in the construction of the microenvironment needed for pre-metastatic niche establishment and metastatic progression [61]. Hence, circulating vesicles are clinically relevant in cancer diagnosis, prognosis and follow up. Exosomes are actually recognised as valid diagnostic tools, but they can also be isolated and exploited as anti-cancer vaccines or nanosized drug carriers in cancer therapy [62].

Nowadays, one of the main issues in cancer diagnosis is the early identification of biomarkers by non-invasive techniques. Obtaining a significant amount of information, before and during tumour treatment, should allow the monitoring of cancer progression and the efficacy of therapeutic regimens. Liquid biopsies to detect circulating tumour cells, RNAs, DNAs and exosomes have been used as indicators for personalised medicine [63]. In recent years, exosomes detection has been validated as a reliable tool for preclinical practice in different cancer types [64], thanks to the identification of their content: double-stranded DNA (dsDNA) [65, 66], messenger RNA (mRNA), micro RNA (miRNA), long non-coding RNA (lncRNA) [67], proteins and lipids [68]. DsDNA has been detected in exosomes isolated from plasma and serum of different cancer cell types, and mutated genes involved in tumorigenesis, such as mutated *KRAS* and *TP53* [69, 70], have been identified as disease predictors. Similarly, exosomal AR-V7 mRNA has been used as a prognostic marker of resistance to hormonal therapy in metastatic prostate cancer patients [71]. Gene expression profiling of multiple RNAs from urinary exosomes has been adopted as an efficient diagnostic tool [72]. LncRNAs isolated from serum exosomes have been exploited for disease prognosis in colorectal cancer patients [73], and multiple miRNAs allow one to distinguish between different lung cancer subtypes [74]. GPC1-positive exosomes have been employed to detect pancreatic cancer [75], while circulating exosomal macrophage migration inhibitory factor (MIF) was able to predict liver metastasis onset [76]. Finally, multiple lipids present in urinary exosomes have been approved as prostate cancer indicators [77]. Due to the high variability of patient classes and sample size, and in order to obtain clinically significant results for a fast and effective diagnosis, huge investments in exosome research will be required in the near future.

Exosomes could also be exploited as natural, biocompatible and low immunogenic nanocarriers for drug delivery in cancer therapy. They can be passively loaded by mixing purified vesicles with small drugs [78–82], or actively loaded by means of laboratory techniques, such as electroporation and sonication [83, 84]. Superparamagnetic nanoparticles conjugated to transferrin have been tested for the isolation of exosomes expressing transferrin receptor from mice blood. After incubation with doxorubicin, they have been used to target liver cancer cells in response to external magnetic fields, inhibiting cell growth both *in vitro* and *in vivo* [80]. Kim *et al.* [83] engineered mouse macrophage-derived exosomes with aminoethyl anisamide-PEG to target sigma receptor, overexpressed in lung cancer cells and passively loaded them with paclitaxel. These systems acted as targeting agents able to suppress metastatic growth *in vivo*.

Three clinical trials with loaded exosomes are currently ongoing for the treatment of different tumours [85–87]: a phase I trial is evaluating the ability of exosomes to deliver curcumin to normal and colon cancer tissues [85]; a phase II trial is investigating the *in vivo* performance of autologous tumour cell-derived microparticles carrying methotrexate in lung cancer patients [86] and a clinical inquiry is focusing on autologous erythrocyte-derived microparticles loaded with methotrexate for gastric, colorectal and ovarian cancer treatment [87].

Recently, new strategies to produce *ad hoc* exosomes have been developed. Cells releasing exosomes have been genetically engineered to overexpress specific macromolecules, or modified to release exosomes with particular targeting molecules [88–90].

Exosomes derived from different cancer cells have already been exploited as cancer vaccines. Autologous dendritic cell-derived exosomes with improved immunostimulatory function have been tested in a phase II clinical trial for the activation of CD8^+^ T cells [91] in non-small cell lung cancer (NSCLC) patients, observing disease stabilisation and a better overall survival [92]. In a phase I trial, ascites-derived exosomes supplemented with granulocyte-macrophage colony stimulating factor (GM-CSF) have been administered to colorectal cancer patients, soliciting a tumour-specific immune response [93].

Many issues related to exosomes clinical translation remain open and are mostly connected to the definition of preclinical procedures for isolation, quantification, storage and standard protocols for drug loading. It is becoming even more necessary to distinguish between tumour and healthy blood cell-derived vesicles to characterise their post-isolation half-life and to perform standard content analyses. For these purposes, innovative approaches and technologies have been set up, such as microarrays and specific monoclonal antibodies and RNA markers amplification strategies [94].

## Natural antioxidants in cancer therapy

Every day, the human body undergoes several exogenous insults, such as ultraviolet (UV) rays, air pollution and tobacco smoke, which result in the production of reactive species, especially oxidants and free radicals, responsible for the onset of many diseases, including cancer. These molecules can also be produced as a consequence of clinical administration of drugs, but they are also naturally created inside our cells and tissues by mitochondria and peroxisomes, and from macrophages metabolism, during normal physiological aerobic processes.

Oxidative stress and radical oxygen species are able to damage DNA (genetic alterations, DNA double strand breaks and chromosomal aberrations [95, 96]) and other bio-macromolecules [97], such as lipids (membrane peroxidation and necrosis [98]) and proteins (significantly changing the regulation of transcription factors and, as a consequence, of essential metabolic pathways [99]).

The protective mechanisms our body has developed against these molecules are sometimes insufficient to counteract the huge damages produced. Recently, in addition to research into the roles of the physiological enzymes superoxide dismutase (SOD), catalase (CAT) and glutathione peroxidase (GP), natural antioxidants such as vitamins, polyphenols and plant-derived bioactive compounds are being studied in order to introduce them as preventive agents and potential therapeutic drugs [100, 101]. These molecules have anti-inflammatory and anti-oxidant properties and are found in many vegetables and spices [102]. Vitamins, alkaloids, flavonoids, carotenoids, curcumin, berberine, quercetin and many other compounds have been screened *in vitro* and tested *in vivo*, displaying appreciable anti-proliferative and pro-apoptotic properties, and have been introduced as complementary therapies for cancer [4, 5, 103].

Despite the advantages of using natural drugs, their translation into clinical practice remains difficult due to their limited bioavailability and/or toxicity. Curcumin, a polyphenolic compound extracted from turmeric (*Curcuma longa*), is a traditional Southeast Asian remedy with anti-inflammatory, anti-oxidant and chemopreventive and therapeutic activities [104]. It has been shown to have cytotoxic effects in different kinds of tumours, such as brain, lung, leukaemia, pancreatic and hepatocellular carcinoma [105, 106], with no adverse effects in normal cells at the effective therapeutic doses [107]. Curcumin can modulate a plethora of cellular mechanisms [108, 109]; however, its biological properties, and as a consequence, the treatment duration and the efficient therapeutic doses, have not been completely elucidated yet. This molecule is highly lipophilic, poorly soluble in water and not very stable [110]. Different strategies and specific carriers, such as liposomes and micelles [111, 112], have been developed to improve its bioavailability. Currently, 24 clinical trials involving curcumin are ongoing and 23 have been already completed [113].

Berberine is an alkaloid compound extracted from different plants, such as *Berberis*. Recently, it has been demonstrated to be effective against different tumours and to act as a chemopreventive agent, modulating many signalling pathways [114, 115]. Like curcumin, it is poorly soluble in water; therefore, different nanotechnological strategies have been developed to facilitate its delivery across cell membranes [116–119]; six clinical trials are open and one has been completed [120].

Quercetin, a polyphenolic flavonoid found in fruits and vegetable, has been proven to be effective to treat several tumours, such as lung, prostate, liver, colon and breast cancers [121–123], by binding cellular receptors and interfering with many signalling pathways [124]. Interestingly, it has been shown to be effective also in combination with chemotherapeutic agents [125]. Presently, seven clinical trials are open and four have been completed [126].

## Targeted therapy and immunotherapy

One of the main problems of conventional cancer therapy is the low specificity of chemotherapeutic drugs for cancer cells. In fact, most drugs act both on healthy and diseased tissues, generating severe side effects. Researchers are putting a lot of effort into finding a way to target only the desired site. Nanoparticles have raised great interest for their tendency to accumulate more in tumour tissues due to the enhanced permeability and retention effect (EPR) [127]. This process, called passive targeting, relies on the small size of nanoparticles and the leaky vasculature and impaired lymphatic drainage of neoplastic tissues [6]. Passive targeting, however, is difficult to control and can induce multidrug resistance (MDR) [128]. Active targeting, on the other hand, enhances the uptake by tumour cells by targeting specific receptors that are overexpressed on them [129, 130]. Nanoparticles, for example, can be functionalized with ligands that univocally bind particular cells or subcellular sites [6]. Several kinds of ligands can be used, such as small molecules, peptides, proteins, aptamers and antibodies.

Folic acid and biotin are small molecules, whose receptors are overexpressed in tumour tissues. Several nanocarriers have been functionalized with folic acid to target ovarian and endometrial cancers [131]: folic acid-conjugated polyethylene glycol-poly(lactic-co-glycolic acid) nanoparticles delivering docetaxel increased drug cellular uptake by human cervical carcinoma cells [132]. Small ligands are cheap and can be linked to nanoparticles by simple conjugation chemistry [133, 134].

Different kinds of small peptides and proteins are also effective in active targeting. Angiopep-2 is a peptide that has raised great interest in the treatment of brain cancer [135], because it binds to low-density lipoprotein receptor-related protein-1 (LRP1) of endothelial cells in the BBB, and it is also overexpressed in glioblastoma cancer cells [136]. Bombesin peptide conjugated to poly(lactic-co-glycolic acid) (PLGA) nanoparticles loaded with docetaxel was used to target the gastrin-releasing peptide receptor, overexpressed on cell surface of prostate, breast, ovarian, pancreatic and colorectal cancer cells [137, 138]. Transferrin is a serum glycoprotein overexpressed on many solid tumours, especially on glioblastoma multiforme cells [139], and on epithelial cells of the BBB [6, 140]. Transferrin-conjugated chitosan-PEG nanoparticles delivering paclitaxel exhibited a higher cytotoxicity towards transferrin-overexpressing human non-small cell lung cancer cells (NSCLCs) (HOP-62) [141].

Aptamers are small synthetic single-stranded RNA or DNA oligonucleotides folded into specific shapes that make them capable of binding specific targets [142]. Farokhzad *et al.* [143] reported that the use of A10 RNA aptamer conjugated to docetaxel-loaded nanoparticles significantly enhances *in vitro* cytotoxicity. The same aptamer has been also used to prepare quantum dot-doxorubicin conjugates [144].

Antibodies are currently the most exploited ligands for active targeting. These proteins have a typical ‘Y’ shape, where the two arms are responsible for the selective interaction with the antigen [145]. Antibodies can be used as immunoconjugates, when conjugated to a drug or nanoparticle, or naked. In the first case, their function is mainly to target a specific antigen overexpressed on cancer cells. Antibodies used for this purpose include those ones that bind to the human epidermal growth factor receptor 2 (HER2), the epidermal growth factor receptor (EGFR), the transferrin receptor (TfR) and the prostate-specific membrane antigen (PSMA) [6]. Rapamycin-PLGA nanoparticle conjugated to EGFR antibody exhibited higher cellular uptake by human breast adenocarcinoma cells (MCF-7), with enhanced apoptotic activity [146]. Loperamide-loaded human serum albumin nanoparticles conjugated to antibodies that specifically bind transferrin receptor successfully crossed the BBB and delivered the drug to the desired site [147].

Naked antibodies or immunoconjugates can also be used in immunotherapy, which is a cancer treatment that aims at stimulating or restoring the immune system of the patient against cancer cells [148]. Antibodies can act as markers for cancer cells to make them more vulnerable to the immune system response (non-specific immune stimulation), or as inhibitors for immune checkpoint proteins on cancer cell surface, that can modulate the action of T-cells [148]. Several antibodies have been already tested and accepted by FDA for immunotherapy, such as rituximab (1997, [149]), ibritumomab tiuxetan (2002, [150]), trastuzumab emtansine (2013, [151]), nivolumab (2014, [152]) and pembrolizumab (2014, [153]).

Immunotherapy can be achieved by another strategy called adoptive cell transfer (ACT) and it consists of isolating T-lymphocytes (T-cells) with the highest activity against cancer directly from the patient’s blood, expanding them *ex vivo*, and reinfusing them again into the patient [154]. Autologous T-cells can be genetically engineered *in vitro* to express a chimaeric antigen receptor (CAR), which makes them more specific against cancer cell antigens [148]. Different CARs can be designed to be directed against a certain cancer antigen. The genetic modification of T-cells can be achieved by different methods such as viral transduction, non-viral methods like DNA-based transposons, CRISPR/Cas9 or other plasmid DNA and mRNA transfer techniques (i.e., electroporation, encapsulation in nanoparticles) [155]. ACT protocols have been already adopted in clinical practice for advanced or recurrent acute lymphoblastic leukaemia and for some aggressive forms of non-Hodgkin’s lymphoma [148]. For example, it has been shown that the treatment of end-stage patients affected by acute lymphocytic leukaemia with CAR T-cells led to a full recovery in up to 92% of patients [155]. Despite these very promising results, much research is currently devoted to understanding the long-term side effects of CAR T-cell therapies and their fate within tumours, and to improving CAR T-cell expansion technologies.

## Gene therapy for cancer treatment

Gene therapy is intended as the introduction of a normal copy of a defective gene in the genome in order to cure specific diseases [156]. The first application dates back to 1990 when a retroviral vector was exploited to deliver the adenosine deaminase (ADA) gene to T-cells in patients with severe combined immunodeficiency (SCID) [157]. Further research demonstrated that gene therapy could be applied in many human rare and chronic disorders and, most importantly, in cancer treatment. Approximately 2,900 gene therapy clinical trials are currently ongoing, 66.6% of which are related to cancer [158]. Different strategies are under evaluation for cancer gene therapy: 1) expression of pro-apoptotic [159, 160] and chemo-sensitising genes [4]; 2) expression of wild type tumour suppressor genes [5]; 3) expression of genes able to solicit specific antitumour immune responses and 4) targeted silencing of oncogenes.

One approach relied on thymidine kinase (TK) gene delivery, followed by administration of prodrug ganciclovir to activate its expression and induce specific cytotoxicity [161]. This has been clinically translated for the treatment of prostate cancer and glioma [162–164]. In recent decades, different vectors carrying the p53 tumour suppressor gene have been evaluated for clinical applications. ONYX-015 has been tested in NSCLC patients and gave a high response rate when administered alone or together with chemotherapy [165]. Gendicine, a recombinant adenovirus carrying wild-type p53 in head and neck squamous cell cancer had a similar success, inducing complete disease regression when combined with radiotherapy [166].

Despite many achievements, there are still some challenges to face when dealing with gene therapy, such as the selection of the right conditions for optimal expression levels and the choice of the best delivery system to univocally target cancer cells. Gene therapy also presents some drawbacks linked to genome integration, limited efficacy in specific subsets of patients and high chances of being neutralised by the immune system. Therefore, particular interest has been elicited by targeted gene silencing approaches.

RNA interference (RNAi) has been recently established as an efficient technology both for basic research and medical translation. Small interfering RNAs (siRNAs) consist of double-stranded RNAs [167] able to produce targeted gene silencing. This process is intracellularly mediated by the RNA-induced silencing complex (RISC), responsible for cleaving the messenger RNA (mRNA), thus leading to interference with protein synthesis [168]. This physiological mechanism has been demonstrated in many eukaryotes, including animals. A few years after RNAi discovery, the first clinical application for wet-age related macular degeneration treatment entered phase I clinical trial [169]. Since cancer is triggered by precise molecular mechanisms, siRNAs can be rationally designed to block desired targets responsible for cell proliferation and metastatic invasion. This strategy relies on siRNA-mediated gene silencing of anti-apoptotic proteins [170], transcription factors (i.e., *c-myc* gene) [171, 172] or cancer mutated genes (i.e., *K-RAS*) [173]. Most of the clinical trials currently ongoing are based on local administration of siRNA oligonucleotides in a specific tissue/organ or on systemic delivery throughout the entire body [9, 174]. Using siRNA-based drugs has several advantages: 1) safety, since they do not interact with the genome; 2) high efficacy, because only small amounts can produce a dramatic gene downregulation; 3) possibility of being designed for any specific target; 4) fewer side effects when compared to conventional therapies and 5) low costs of production [175, 176]. However, siRNAs are relatively unstable *in vivo* and can be phagocytosed during blood circulation, excreted by renal filtration, or undergo enzymatic degradation [177]. Occasionally, they can induce off-target effects [178] or elicit innate immune responses, followed by specific inflammation [179, 180]. Since naked siRNAs are negatively charged hydrophilic molecules, they cannot spontaneously cross cell membranes. Consequently, different delivery strategies are currently under study, such as chemical modification, encapsulation into lipid or polymeric carriers or conjugation with organic molecules (polymers, peptides, lipids, antibodies, small molecules [181], for efficient targeting [182, 183]). Chemical modifications include the insertion of a phosphorothioate at 3’ end to reduce exonuclease degradation [184], the introduction of 2’ O-methyl group to obtain longer half-life in plasma [185] and the modification by 2,4-dinitrophenol to favour membrane permeability [186]. Nevertheless, the degradation of modified siRNAs often elicits cytotoxic effects; therefore, it is preferable to design *ad hoc* nanocarriers.

Different cationic lipid nanoparticles, such as liposomes, micelles and solid lipid nanoparticles [183], have been exploited for siRNA loading. Cationic liposomes interact with negatively charged nucleic acids, which can be easily transfected by simple electrostatic interactions [187, 188]. They can be constituted by 1,2-dioleoyl-3-trimethylammonium propane (DOTAP) and N-{1-(2,3-dioleoyloxy) propyl]-N,N,N-trimethylammonium methyl sulphate (DOTMA) [189]. A theranostic agent consisting of an anticancer survivin siRNA entrapped in PEGylated liposomes has been developed to achieve simultaneous localisation inside tumour cells by means of entrapped MR agents and fluorophores and reduction of proliferation *in vivo* [190].

Neutral liposomes based on 1,2-dioleoyl-sn-glycero-3-phosphatidylcholine (DOPC) have shown high efficacy in mice models of ovarian carcinoma and colorectal cancer [191, 192]. A phase I clinical trial is currently recruiting patients for evaluating the safety of siRNA-EphA2-DOPC when administered to patients with advanced and recurrent cancer [193].

Stable nucleic acid lipid particles (SNALPs) have been evaluated in non-human primates [194]. SiRNAs have been encapsulated in a mixture of cationic lipids coated with a shell of polyethylene glycol (PEG) [195]. SNALPs entered a phase I clinical trial in patients affected by advanced solid tumours with liver involvement [196] and a phase I/II trial for treating neuroendocrine tumours and adrenocortical carcinoma patients refractory to standard therapy [197].

SiRNAs can be condensed in cationic polymers such as chitosan, cyclodextrin and polyethylenimine (PEI). Chitosan is a natural polysaccharide that, due to its cationic charge, has been exploited as carrier for nucleic acids *in vitro* and *in vivo* [198]. Specifically, a targeted siRNA has been delivered in mice xenografts of breast cancer [199]. Cyclodextrin polymers coated with PEG, conjugated with human transferrin and carrying a siRNA called CALAA-01, inhibit tumour growth by reducing the expression of M2 subunit of ribonucleotide reductase (R2), and have entered a phase I clinical trial [200]. PEI is able to form small cationic nanoparticles containing siRNAs and it has been exploited as antitumoural, upon loading with HER-2 receptor-specific siRNA [201]. A phase II clinical trial is presently starting to evaluate siG12D LODER directed to mutated KRAS oncogene and encapsulated into a biodegradable polymeric matrix for locally treating advanced pancreatic cancer patients in combination with chemotherapy [202].

SiRNAs may be conjugated to peptides, antibodies and aptamers in order to improve their stability during circulation and to enhance cellular uptake [203]. A success is represented by siRNAs targeting PSMA, overexpressed in this type of cancer [204].

The introduction of nanocarriers has largely improved siRNAs stability, pharmacokinetics and biodistribution properties, and the targeting specificity [205, 206]. Smart nanomaterials responsive to external (i.e., magnetic field, ultrasounds) and tumour-specific stimuli (i.e., acidic pH, redox conditions) are currently under the development for controlled release and reduction of undesired negative effects [207, 208]. Nanocarriers delivering siRNAs undergo a series of pH variations from blood circulation to intracellular environment and, for this reason, many pH responsive materials have been designed to favour cargo release under specific pH conditions [209]. Poly(allylamine) phosphate nanocarriers, stable at physiological pH, have been developed to release siRNAs in the cytoplasm after disassembly at low endosomal pH [210].

Although there have been many successes, some questions remain open and make the clinical translation of the siRNA-based approach very challenging, such as the correct doses to be delivered to patients and the many variabilities observed between individuals and different stages of disease. Further research towards controlled release to reach only specific targets, and the set-up of the best personalised therapy for cancer patients will be necessary in the near future.

## Thermal ablation and magnetic hyperthermia

Thermal ablation of tumours includes a series of techniques that exploit heat (hyperthermia) or cold (hypothermia) to destroy neoplastic tissues [13]. It is known that cell necrosis occurs at temperatures lower than -40°C or higher than 60°C. Long exposures to temperatures between 41°C and 55°C are also effective for tumour cell damage. Moreover, it has been shown that cancer cells are more sensitive to high temperatures than healthy ones [211].

Hypothermic ablation is due to the formation of ice crystals upon cooling, which destroy cell membranes and finally kill cells. Argon gas is the preferred cooling agent because it can cool down the surrounding tissues to -160°C. Also, gases at their critical point, such as nitrogen, can be exploited since they have a higher heat capacity than argon. However, the technology to control and direct them is not well developed yet [10].

Hyperthermic ablation currently comprises radiofrequency (RF), microwave and laser ablation [10].

RF ablation is the most used in clinics, because it is effective and safe [212]. An alternated current of RF waves is applied to a target zone by an insulated electrode tip, while a second electrode, needed to close the circuit, is placed on the skin surface [10]. The interaction with the current causes the oscillation of ions in the extracellular fluid, which, in turns, produces heat. The more conductive the medium, the more effective the process. For this reason, RF ablation works very well in the liver and in other areas with a high content of water and ions, whereas it has a poor effect in lungs [10]. Moreover, the efficiency of the treatment decreases with the size of the lesion, giving the best results for areas not larger than 3 cm^2^ [213, 214].

Microwave ablation is based on the electromagnetic interaction between microwaves and the polar molecules in tissues, like water, that causes their oscillation and the consequent increase in temperature. Unlike the electrical current in RF ablation, microwaves can propagate through any kind of tissue [215, 216], and this allows high temperatures to be reached in a short amount of time, to have a deeper penetration and to treat larger areas of tumours [217].

Laser therapy exploits the properties of laser beams of being very narrow and extremely focused at a specific wavelength. This makes the treatment very powerful and precise, thus a promising alternative to conventional surgery [218]. The absorption of the light emitted by the laser results in the heating and subsequent damage of the treated area [219]. Depending on the specific application, different kinds of lasers can be used. Neodymium:yttrium-aluminium-garnet (Nd:YAG) lasers (wavelength of 1064 nm) and diode lasers (wavelength of 800–900 nm) are used to treat internal organs, since they have a penetration depth up to 10 cm [218]. Conversely, CO_2_ lasers (10,600 nm), with a penetration depth of 10 μm up to 1 mm maximum are used for superficial treatments. Laser therapy is receiving a lot of attention in research because of its advantages compared to other ablation techniques, such as a higher efficacy, safety and precision, and a shorter treatment session needed to achieve the same results [220, 221]. Moreover, the fibres to transmit laser light are compatible with MRI, allowing for a precise measure of the temperature and the thermal dose [222]. However, there are still some limitations to overcome, such as the need of a very skilled operator to place the fibre in the correct position [218].

Finally, a new way to heat tumour tissues, currently under study, is through magnetic hyperthermia. This technique exploits superparamagnetic or ferromagnetic nanoparticles that can generate heat after stimulation with an alternating magnetic field. The most studied systems in nanomedicine are SPIONs [11]. The production of heat, in this case, is due to the alignment of magnetic domains in the particles when the magnetic field is applied, and the subsequent relaxation processes (Brownian and/or Neel relaxations) during which heat is released, when the magnetic field is removed and the magnetisation of the particles reverts to zero [223]. Magnetic hyperthermia can reach any area of the body and SPIONs can also act as MRI contrast agents to follow their correct localisation before the stimulation. The particles can be coated with biocompatible polymers and/or lipid and functionalized with specific ligands to impart targeting properties [224]. As already mentioned, until now, just a formulation of 15-nm iron oxide nanoparticles coated with aminosilane (Nanotherm) obtained approval for the treatment of glioblastoma [31]. SPIONs have also been successfully encapsulated in lipid nanocarriers together with a chemotherapeutic agent to combine chemotherapy and hyperthermia [49, 50].

## Recent innovations in cancer therapy: Radiomics and pathomics

Efficient cancer therapy currently relies on surgery and, in approximately 50% of patients, on radiotherapy, that can be delivered by using an external beam source or by inserting locally a radioactive source (in this case, the approach is named brachytherapy), thus obtaining focused irradiation. Currently, localisation of the beam is facilitated by image-guided radiotherapy (IGRT), where images of the patient are acquired during the treatment allowing the best amount of radiation to be set. Thanks to the introduction of intensity-modulated radiotherapy (IMRT), radiation fields of different intensities can be created, helping to reduce doses received by healthy tissues and thus limiting adverse side effects. Finally, by means of stereotactic ablative radiotherapy (SABR), it has become feasible to convey an ablative dose of radiation only to a small target volume, significantly reducing undesired toxicity [225].

Unfortunately, radioresistance can arise during treatment, lowering its efficacy. This has been linked to mitochondrial defects; thus, targeting specific functions have proven to be helpful in restoring anti-cancer effects [226]. A recent study has shown, for example, that radioresistance in an oesophageal adenocarcinoma model is linked to an abnormal structure and size of mitochondria, and the measurement of the energy metabolism in patients has allowed discrimination between treatment resistant and sensitive patients [227]. Targeting mitochondria with small molecules acting as radiosensitizers is being investigated for gastrointestinal cancer therapy [228].

Cancer is a complex disease and its successful treatment requires huge efforts in order to merge the plethora of information acquired during diagnostic and therapeutic procedures. The ability to link the data collected from medical images and molecular investigations has allowed an overview to be obtained of the whole tridimensional volume of the tumour by non-invasive imaging techniques. This matches with the main aim of precision medicine, which is to minimise therapy-related side effects, while optimising its efficacy to achieve the best individualised therapy [229].

Radiomics and pathomics are two promising and innovative fields based on accumulating quantitative image features from radiology and pathology screenings as therapeutic and prognostic indicators of disease outcome [12, 13, 230]. Many artificial intelligence technologies, such as machine learning application, have been introduced to manage and elaborate the massive amount of collected datasets and to accurately predict the treatment efficacy, the clinical outcome and the disease recurrence. Prediction of the treatment response can help in finding an *ad hoc* adaptation for the best prognosis and outcome. Nowadays, personalised medicine requires an integrated interpretation of the results obtained by multiple diagnostic approaches, and biomedical images are crucial to provide real-time monitoring of disease progression, being strictly correlated to cancer molecular characterisation.

Radiomics is intended as the high throughput quantification of tumour properties obtained from the analysis of medical images [14, 15, 231]. Pathomics, on the other side, relies on generation and characterisation of high-resolution tissue images [16, 232, 233]. Many studies are focusing on the development of new techniques for image analysis in order to extrapolate information by quantification and disease characterisation [234, 235]. Flexible databases are required to manage big volumes of data coming from gene expression, histology, 3D tissue reconstruction (MRI) and metabolic features (positron emission tomography, PET) in order to identify disease phenotypes [236, 237].

Currently, there is an urgent need to define univocal data acquisition guidelines. Some initiatives to establish standardised procedures and facilitate clinical translation have been already undertaken, such as quantitative imaging network [238] or the German National Cohort Consortium [239]. Precise description of the parameters required for image acquisition and for the creation and use of computational and statistical methods are necessary to set robust protocols for the generation of models in radiation therapy. According to the US National Library of Medicine, approximately 50 clinical trials involving radiomics are currently recruiting patients, and a few have already been completed [240].

## Conclusions and future perspectives

**Figure 1.**
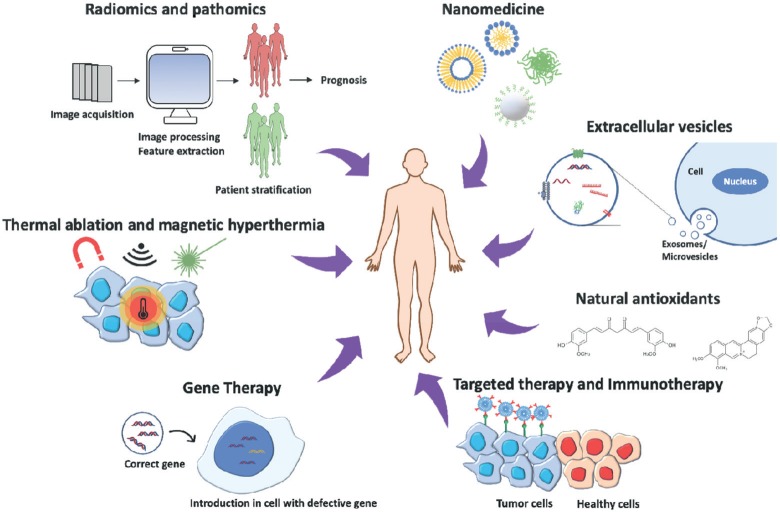
Cancer therapy approaches: The image represents the most innovative strategies to treat cancer, combining different disciplines to obtain the most efficient and personalised therapy for patients.

In recent years, research into cancer medicine has taken remarkable steps towards more effective, precise and less invasive cancer treatments (Figure 1). While nanomedicine, combined with targeted therapy, helped improving the biodistribution of new or already tested chemotherapeutic agents around the specific tissue to be treated, other strategies, such as gene therapy, siRNAs delivery, immunotherapy and antioxidant molecules, offer new possibilities to cancer patients. On the other hand, thermal ablation and magnetic hyperthermia are promising alternatives to tumour resection. Finally, radiomics and pathomics approaches help the management of big data sets from cancer patients to improve prognosis and outcome.

**Figure 2.**
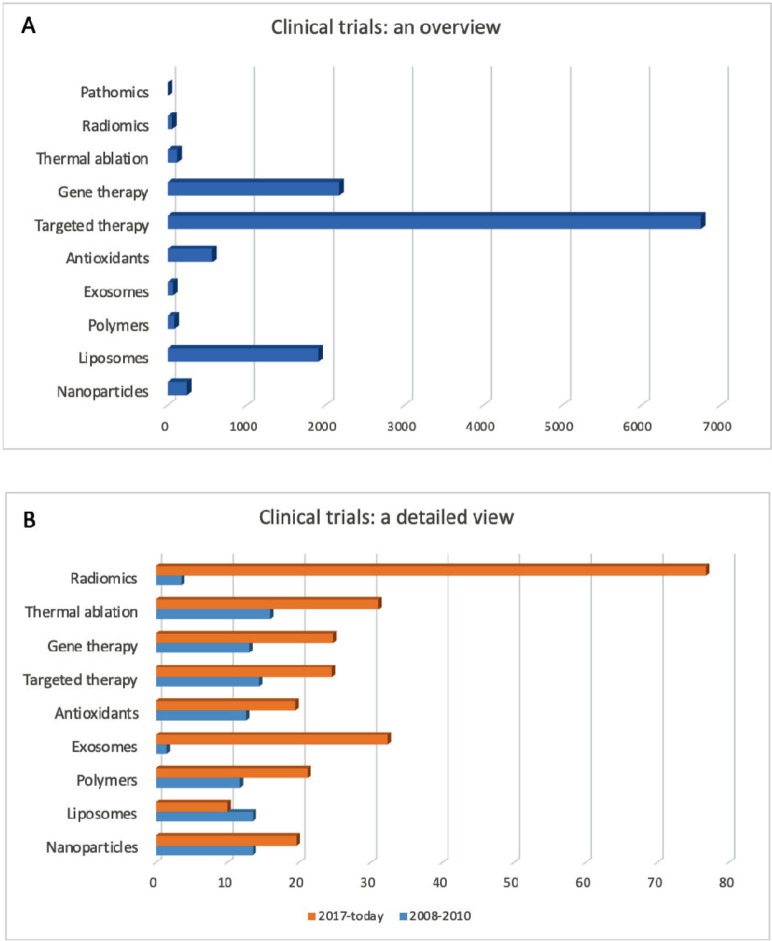
Cancer clinical trials. (A): Total number of clinical trials currently registered on www.clinicaltrials.gov for each approach discussed in this review. (B): Number of the clinical trials [in % respect with the total studies shown in (A)] started during the years 2008–2010 (blue) and from 2017 until today (orange). Date accessed: 01/08/19

At the moment, the most frequent entries concerning cancer therapies in the database of clinical trials (www.clinicaltrials.gov) involve the terms targeted therapy, immunotherapy and gene therapy, highlighting that these are the most popular methodologies under investigation, especially because, as already mentioned before, they have been shown to be very promising and effective (Figure 2A). However, Figure 2B shows that the clinical trials started in the past decade on different therapies mentioned in this review (except for liposomes-based therapies) have increased in number, showing how the interest on these new approaches is quickly growing in order to replace and/or improve conventional therapies. In particular, radiomics, immunotherapy and exosomes are the entries whose number has increased the most in the last 10 years.

The current scenario for cancer research is wide, offering many possibilities for the constant improvement of treatment, considering not only patient recovery but also caring for their well-being during therapy. As summarised in Table 1, these new approaches offer many advantages compared to conventional therapies. However, some disadvantages still have to be overcome to improve their performances. Much progress has been made, but many others are likely to come in the near future, producing more and more *ad hoc* personalised therapies.

## Conflicts of interest

The authors declare that they have no conflict of interest.

## Funding declaration

This work was partially supported by the Fondazione CaRiPLo, grant no. 2018-0156 (Nanotechnological countermeasures against Oxidative stress in muscle cells Exposed to Microgravity—NOEMI) and by the European Research Council (ERC) under the European Union’s Horizon 2020 Research and Innovation Programme (grant agreement N°709613, SLaMM).

## Authors’ contributions

Carlotta Pucci and Chiara Martinelli contributed equally to this work.

## Figures and Tables

**Table 1.** Advantages and disadvantages of the main innovative cancer therapeutic approaches.

Strategy	Advantages	Disadvantages
Nanoparticles	• High stability and specificity • Good biocompatibility and bioavailability	• It depends on the particular nanoparticle
EVs	• Physiologically secreted • Good molecular characterisation • High biocompatibility • *In vitro* modifiable/loadable	• Lack of preclinical procedures for isolation, quantification, storage and drug loading
Natural antioxidants	• Easily available in large quantities • Exploitation of their intrinsic properties	• Limited bioavailability • Possible toxicity
Targeted therapy	• High specificity • Reduction of adverse reactions	• Lack of information regarding long-term side effects
Gene therapy	• Expression of pro-apoptotic and chemo-sensitising genes • Expression of wild type tumour suppressor genes • Expression of genes able to solicit specific anti-tumour immune responses • Targeted silencing of oncogenes and safety (RNAi)	• Genome integration • Limited efficacy in specific subsets of patients • High chances to be neutralised by immune system • Off-target effects and inflammation (RNAi) • Need of *ad hoc* delivery systems (RNAi) • Set-up of doses and suitable conditions for controlled release (RNAi)
Thermal ablation Magnetic hyperthermia	• Precise treatment of the interested area • Possibility to perform the treatment along with MRI imaging (magnetic hyperthermia)	• High efficiency only for localised areas • Low penetration power • Need for a skilled operator to perform the treatment
Radiomics/pathomics	• Creation of tumour whole tridimensional volume by non-invasive imaging techniques • Therapeutic and prognostic indicators of disease outcome	• Definition of univocal data acquisition guidelines • Standardisation of procedures to facilitate clinical translation • Description of parameters and computational/statistical methods to set robust protocols for the generation of models for therapy
